# Surgery

**Published:** 1893-11

**Authors:** 


### SURGERY.

EDITED BY DR. F. W. MCRAE.

### A NEW AND RAPID METHOD OF ANESTHESIA.

As the first American to employ this method, it may be permitted me to invite the attention of American surgeons to this subject by this brief notice.

The method was devised and developed, during the summer and fall of 1892, by Dr. Bourbon, anesthesist of Hôpital Bichat, Dr. Hartman, acting surgeon, and myself, and, since its introduction, is the preferred method in the surgical service of Professor Terrier, at Hôpital Bichat. The *modus operandi* cannot be better described briefly than is done by Professor Terrier in his communiction of the subject to the Société de Chirurgie of Paris, October 19, 1892.

"The bromide of ethyl should be given in large doses. After pouring it abundantly (about three grammes) upon a folded towel, it is placed over the nose and mouth of the patient, who is told to breathe deeply. Generally at the second or third inhalation a slight agitation appears, but at the fifth or sixth the anesthesia is complete, with total loss of consciousness. With the continuance of this anesthetic, in a moment, sometimes preceded by tonic contracture, the complete muscular resolution is accomplished, with congested face and dilated pupil. At this moment the towel saturated with ethyl bromide is rejected, and another upon which chloroform is poured, is substituted in its place, without allowing any interruption of anesthesia in changing. The first dose of chloroform only should be fairly strong (one gramme). At this moment the operation may be commenced (about three-quarters to one minute since the commencement of anesthesia). The facial congestion diminishes little by little, the pupilary dilatation gives place to retraction; in a word, the transition from the anesthesia of the bromide to that of chloroform is accomplished without the slightest interruption of the sleep. From this moment the continuance of the anesthesia is done in the ordinary way (chloroform in small and regular doses). The difference is simply that the complete anesthesia is immediate, instead of resultant of a period often long, which is necessary in the process of chloroform anesthesia."

It will be seen that the duration of the anesthesia may be prolonged at the will of the operator. Experience has demonstrated that by this process a remarkably small quantity of anesthetic agents suffices for complete and protracted unconsciousness, and that by reason of the smallness of the quantity used, the awakening of the patient almost immediately follows the cessation of administering the chloroform.

I have performed a complete anesthesia for ovariotomy by Professor Terrier, with the administation of three grammes of ethyl bromide and twelve grammes of chloroform, recovery from sleep immediately, and no subsequent nausea. Duration of the complete anesthesia thirty-two minutes.

This is only one of many similar cases which will be found in the publication of the clinical studies. How many anesthesists are there who can obtain a complete resolution of a vigorous subject with twelve grammes of chloroform only?

The advantages claimed for this method are:

1. Rapidity, a complete anesthesia, which allows the commencement of the operation in one minute's time after the first inhalation of the anesthetic.

2. Doing away with all violent agitation on the part of the patient, so common when chloroform or ether alone is used.

3. On account of the dissimilar physiological action of the two anesthetics employed, and the dangers attributed to the use of chloroform alone are in a great measure eliminated by the counteraction of the ethyl bromide previously administered.

4. An almost entire absence of post-operatory nausea.

In February of this year this method was introduced in the service of Dr. Segond, at the Maison de Santé, the City Hospital of Paris, by Dr. Malherbe, and immediately adopted by Dr. Segond for all of his work, to the exclusion of other methods. It was my privilege to introduce this method in the service of Dr. Monod, at Hôpital St. Antoine, in April of this year, which led to its adoption as the current method throughout his wards. Dr. Richelot, of Hôpital St. Louis, has spoken with considerable favor of this process of anesthesia, in the discussion at the Société de Chirurgie, and I am imformed that in his service he employs it in preference to all others, in cases where affections of the heart cause any apprehension from the use of chloroform alone.

Convinced of its general utility, I would invite its trial in the work of American surgeons, that it may be widely known, experimented, and thoroughly studied and discussed.—*Dr. W. S. Magill, in Medical Record.*

---

### THE USE OF SALINES IN APPENDICITIS.

The theoretical action of cathartics in peritonitis, as given by various men, consists in an absorption and removal by intestinal drainage of the toxic products of certain micro-organisms which, multiplying in or near the peritoneal cavity, endanger life. I do not object to carrying out this theory after the appendix has been securely tied, or after it is clear that there is no danger of rapid extravasation; but in the first forty-eight hours of appendicitis I look upon the administration of salines as extremely dangerous, and as a not infrequent cause of general peritonitis and death. The reasons for this lie in the pathological conditions that exist in a very considerable percentage of cases. If in a given case there is a perforation in an appendix of large lumen, salines, by liquefying the feces and increasing peristalsis *will cause an immediate and almost invariably fatal extravasation.* In such a pathological condition, which is not infrequent, the use of cathartics before removal and ligation of the appendix must be and is attended by most fatal consequences.

There is the same objection to the use of salines in gun-shot wounds of the intestines, in perforations of typhoid fever, or in perforating ulcers of the intestinal tract generally.

In catarrhal appendicitis, without perforation—the mildest of diseases—there is no hurry to produce catharsis; should the perforation suddenly occur, by keeping the bowels quiet you avoid the danger of rapid extravasations, and give the surgeon a chance to remove the appendix before a general peritonitis makes the outlook hopeless. In a localized peritonitis, if no surgical interference has been made, the recent adhesions may be ruptured by fresh extravasations forced along by a stimulated peristaltic *vis-a-tergo,* and a general peritonitis quickly develop.

These objections to the administration of salines are based upon a very considerable experience. I may say that a majority of the fatal cases of appendicitis have been attended by extensive fecal extravasations. I do not mean to assert that these extravasations have been caused in every instance by the administration of saline cathartics; but I do mean to state most unequivocally that they have been so caused in many cases, and that, furthermore, the existing conditions of general spreading peritonitis, far from being curable by salines, are, in my opinion and experience, beyond relief even by the most radical surgical measures, except in very rare instances. Moreover, in these extraordinary cases where, in a fully established peritonitis, recovery, with or without operation, takes place, I believe we shall find that only comparatively innocuous micro-organisms are present; on the other hand, the explanation of certain fulminating cases, even if apparently trivial at the outset, lies in the presence and rapid reproduction of germ colonies of great vitality and virulence. In either case, I am convinced, the action of saline cathartics has very little to do with the result; except in certain instances, for the reasons above given to hasten the fatal end.

If the appendix has been tied off, or if the peritoneal cavity has been walled off with gauze, or if there is a firmly localized peritonitis, I do not object to cathartics, and I use the salines freely. I must say, however, that in a completely established general peritonitis, from whatever cause, with distention, vomiting and obstipation, in my experience, salines accomplish absolutely nothing.

To produce "intestinal drainage" after abdominal operations I think salines most excellent; and they have their use in the very beginning of a peritonitis in which there is no question of extravasation. I believe the future use of salines will be confined to these conditions.—Dr. M. H. Richardson in *Boston Medical and Surgical Journal*.

---

### NEW METHOD OF DRAINAGE IN SUPRAPUBIC CYSTOTOMY.

Farrar Cobb (*Boston Medical and Surgical Journal*) describes a method which he adopted in a case of suprapubic cystotomy, where it was difficult to drain the bladder well. An ordinary glass drainage tube round at the lower end, with a collar at the top, was passed into the bladder, and into this was pushed for its whole length a tight roll of iodoform gauze, whilst the free end of this, about three feet long, was drawn through a flexible rubber tube, and led over the side of the bed into a bottle. By this means capillary drainage was established. The wound was then packed with absorbent cotton wool thoroughly impregnated with oxide of zinc ointment, which acts as an efficient drain and antiseptic dressing, and prevents any irritation of the skin. Iodoform gauze can not be placed in the wound owing to its power of absorbing the urine. Over the dressing of wool and ointment a layer of gauze may be placed. By this means the patient was kept dry for twelve hours at a time, and only required dressing once or twice a day, the roll of gauze in the tube being changed once every twenty-four hours. After dressing the wound it is advisable to draw off with a syringe any urine which may be present in the bladder. The patient upon whom the above method was adopted previously complained of pain and tenesmus, which soon afterwards disappeared.

